# Fermentation of Whole-Wheat Using Different Combinations of Lactic Acid Bacteria and Yeast: Impact on In Vitro and Ex Vivo Antioxidant Activity

**DOI:** 10.3390/foods14030421

**Published:** 2025-01-28

**Authors:** Elena Tomassi, Nafiou Arouna, Milena Brasca, Tiziana Silvetti, Sabrina de Pascale, Antonio Dario Troise, Andrea Scaloni, Laura Pucci

**Affiliations:** 1 Institute of Agricultural Biology and Biotechnology, National Research Council, 56124 Pisa, Italy; elena.tomassi@ibba.cnr.it; 2 Department of Agricultural Sciences, University of Naples Federico II, 80055 Portici, Italy; nafiou.arouna@unina.it; 3 Institute of Sciences of Food Production (ISPA), Italian National Research Council, 20133 Milan, Italy; milena.brasca@ispa.cnr.it (M.B.); tiziana.silvetti@ispa.cnr.it (T.S.); 4 Institute for the Animal Production System in the Mediterranean Environment, National Research Council, 80055 Portici, Italy; sabrina.depascale@cnr.it (S.d.P.); antoniodario.troise@cnr.it (A.D.T.); andrea.scaloni@cnr.it (A.S.)

**Keywords:** fermentation, lactic acid bacteria co-cultures, yeast, antioxidant, whole-wheat, short-chain fatty acids

## Abstract

Cereals are rich in nutrients and bioactive compounds; however, many of these, such as polyphenols, are bound to the cell wall matrix, limiting their bioavailability. This study investigated the use of fermentation to enhance the bioavailability of functional compounds in whole-wheat flour. Given the impact of microbial species on fermentation outcomes, various combinations of lactic acid bacteria and yeast strains were examined. The polyphenol and flavonoid content of different fermented flours was analyzed. Additionally, the antioxidant capacity was assessed using in vitro assays (DPPH, ORAC, and FRAP) and an ex vivo test with human erythrocytes. Fermentation significantly enhanced the release of bioavailable phenolic compounds and flavonoids, with the most significant increases reaching up to 3.4-fold and 2.64-fold, respectively. In particular, the findings highlight the capacity of flour fermented with a combination of *K. humilis*, *F. sanfranciscensis*, *E. faecium*, *P. pentosaceus*, and *L. mesenteroides* to enhance antioxidant activity in vitro and to protect human red blood cells from oxidative stress. Furthermore, fermentation increased the production of short-chain fatty acids, notably lactate and acetate, which are widely recognized for their gut health benefits. Overall, this study highlights the effectiveness of targeted fermentation in improving the bioactivity and antioxidant properties of whole-wheat flour.

## 1. Introduction

Cereals are a good source of fiber, proteins, minerals, and bioactive components. Among cereals, wheat (*Triticum aestivum*) is the most widely consumed [1,2]. Whole-wheat has notable nutritional and nutraceutical properties, consisting of approximately 55% *w*/*w* carbohydrates, with 50–80% of the bran’s outer layer made up of minerals (zinc, copper, iron, and magnesium), antioxidants (polyphenols and specifically anthocyanins), B vitamins, and fiber [3,4]. However, most of the bioactive compounds, including polyphenols, are primarily found to be covalently linked through ester or ether bonds to cell wall components, such as arabinoxylans and lignin [5,6].

Dietary fiber and antioxidant compounds, mainly polyphenols, are closely associated in their physiological roles. Around 50% of total dietary antioxidants are bound to dietary fiber as they pass through the small intestine. In the large intestine, the gut microbiota actively contributes to fiber degradation, leading to the release of polyphenols [7].

Fermentation is a process in which microorganisms break down organic molecules into simpler compounds, resulting in the biosynthesis and transformation of bioactive compounds. Fermentation also enhances the bioavailability of cereal minerals by degrading phytates, which are abundant in whole grains, through the activity of specific lactobacilli strains [3,8]. During fermentation, lactic acid bacteria (LAB) produce a wide range of metabolites, including organic acids and exopolysaccharides (EPSs), which have anti-inflammatory and antioxidant properties [9,10,11,12]. As natural sources of antioxidants, fermented foods have gained increasing attention for their ability to protect against oxidative damage and promote human health [13].

However, the characteristics of fermented foods are largely determined by factors such as the type of substrates, the specific strain of microorganisms used, the synergy among the microorganisms in co-cultures, and the duration of fermentation, with longer times being generally preferable to achieve greater changes [14]. Common fermenting microorganisms include LAB genera such as *Enterococcus, Streptococcus*, *Leuconostoc, Lactobacillus*, and *Pediococcus*, along with various yeasts, including *Saccharomyces* and *Kazachstania*. These microorganisms not only improve food quality but also enhance food safety by inhibiting harmful compounds and pathogens [15,16]. Fermentation of whole-wheat flour is often carried out using sourdough microbiota, which is mainly composed of LAB with counts of 10^8^ to 10^9^ CFU per gram of sourdough. In general, LAB, such as *Fructilactobacillus sanfranciscensis* and *Lactiplantibacillus plantarum*, are the predominant microorganisms involved in sourdough fermentation, while yeasts often coexist in significant numbers [17].

Furthermore, fermentation and microbial metabolism streamline the production of short-chain fatty acids (SCFAs) during both food processing and large-intestine transit. Microorganisms, such as LAB and yeasts, produce organic acids like lactate and other α-hydroxy acids, which act as precursors for SCFA synthesis by gut microbiota. SCFAs, including acetate, propionate, and butyrate, play critical roles in maintaining gut health by supporting epithelial integrity, optimizing signaling, reducing inflammation, and serving as an energy source for colon cells [18]. Additionally, fermented foods modulate gut microbiota by promoting the growth of SCFA-producing bacteria, such as *Faecalibacterium prausnitzii* and *Eubacterium rectal* [19,20].

This study aimed to provide insights into the effect of microbial blends on the fermentation of whole-wheat flour to improve its nutritional and antioxidant value. Specifically, we formulated several microbial mixtures containing yeast and lactic acid bacterial strains from different food sources. The selected bacterial species were chosen based on research evidence supporting their ability to enhance nutritional value, including improving protein digestibility, increasing nutrient bioavailability, and degrading anti-nutritional factors. Additionally, species such as *L. plantarum* and *Pediococcus pentosaceus* were selected for their antioxidant properties [21,22]. Lactic acid bacteria, such as *Enterococcus faecium* and *Streptococcus thermophilus*, were selected for their proteolytic activity, which enables the generation of bioactive peptides with antioxidant activity, as well for their suitability as starter cultures for food functionalization [23,24].

## 2. Materials and Methods

### 2.1. Chemicals and Reagents

All standards and reagents were of analytical grade. Folin–Ciocalteu reagent, gallic acid, sodium carbonate, phosphate-buffered saline (PBS), 2,4,6-Tri(2-pyridyl)-s-triazine (TPTZ), ethylenediamine-tetraacetic acid (EDTA), acetic acid, 2,2-diphenyl-1-picrylhydrazyl (DPPH), 2,2-azobis (2-amidinopropane) dihydrochloride (AAPH), fluorescein sodium salt, and 2′,7′-dichlorofluorescein diacetate (DCFH-DA) were purchased from Sigma-Aldrich (St. Louis, MA, USA), while pyridine was obtained from Fisher Scientific (Bremen, Germany). Water, methanol, and acetonitrile were of mass spectrometry grade and were obtained from Merck (Darmstadt, Germany). The derivatizing agent for SCFA quantification, 3-nitrophenyhydrazine (3-NPH), N-(3-dimethylaminopropyl)-N′-ethylcarbodiimide (EDC), quinic acid, and all the analytical standards including sodium 3-hydroxybutyrate, butyric acid, isobutyric acid, acetic acid, lactic acid, propionic acid, valeric acid, isovaleric acid, ^13^C_2_-acetic acid, ^13^C_3_-propionic acid, and ^13^C_4_-butyratic acid were purchased from Sigma-Merck (Darmstadt, Germany).

### 2.2. Preparation of Lactic Acid Bacteria and Yeast Inocula

*Kazachstania humilis* (ITEM 19255) and *Saccharomyces cerevisiae* were grown in Yeast Malt broth (Scharlau Microbiology, Barcelona, Spain) at room temperature without shaking under aerobic conditions, whereas lactic acid bacteria were cultivated using de Man, Rogosa, and Sharpe (MRS) broth with Tween 80 (Biolife Italiana, Milan, Italy) at 30 °C (*L. plantarum* VS513 and *Leuconostoc mesenteroides* GP19) or 37 °C (*P. pentosaceus* ITEM 18337), maltose (Biolife Italiana) MRS broth at 30 °C (*Fructilactobacillus sanfranciscensis* ITEM 19254) according to Vera et al. [25], and M17 broth (Biolife Italiana) at 37 °C (*Streptococcus thermophilus* E and *Enterococcus faecium* ITEM 19253). All strains were aerobically incubated in ambient air overnight, except for *F. sanfranciscensis,* which was incubated under anaerobic conditions (Anaerocult A, Merck, Darmstadt, Germany), and sub-cultured twice in their respective media before use.

The strains were obtained from the bacterial collection of the ISPA-CNR (Institute of Science of Food Production of the Italian National Research Council, Milan, Italy). *K. humilis*, *S.s cerevisiae*, *F. sanfranciscensis*, and *S. thermophilus* were isolated from sourdough, while the other strains were derived from different sources (wheat bran, beer, and dairy samples). Bacterial strains were identified through partial 16S rRNA sequencing using the universal primer set p8FPL and p806R [26]. Yeasts were identified by sequencing the D1/D2 domain of the 26S rDNA gene according to Kurtzman and Robnett [27] and/or the Internal Transcribed Spacer (ITS) region according to Schoch et al. [28] (Table S1). Cleaned-up PCR products were sent to Macrogen Europe (Amsterdam, the Netherlands) for sequencing, and the resulting sequences were analyzed with NCBI (http://www.ncbi.nlm.nih.gov/BLAST, accessed on 22 December 2021) and MycoBank (http://www.mycobank.org/, accessed on 22 December 2021) databases.

As for the *E. faecium* strain ITEM 19253, its safety of use was evaluated according to the current criteria provided by EFSA [29].

### 2.3. Whole-Wheat Fermentation Conditions and Extraction of Bioactive Compounds

For the fermentation process, commercial whole-wheat flour (600 g) was mixed with 900 mL of water in a HotmixPRO Gastro (HotMix, Modena, Italy) incubator, which ensured a continuous flow of air and a constant temperature of 37 °C. Then, for the different inocula (Mix 1–Mix 8, as detailed in Table 1), an equal volume of each single LAB or yeast strain, containing 10⁸ cells and 10⁶ cells, respectively, was added simultaneously to the dough. Fermentation was carried out with continuous mixing for four days (96 h).

After 96 h, the samples were collected and stored at −80 °C. The frozen samples were then lyophilized for three days using a freeze-dryer Lyovac GT 2 (SRK Systemtechnik, Riedstadt, Germany).

Bioactive compounds were extracted from fermented and unfermented whole-wheat flour by suspending the samples in double-distilled water at a concentration of 50 mg/mL. The suspension was shaken for 2 h and then centrifuged at 2140 rcf for 10 min, at 4 °C, using the Jouan CR 3i centrifuge. The supernatant was collected and stored at 4 °C until further use. Extraction was carried out in triplicate.

### 2.4. Determination of Total Phenolic and Flavonoid Content

The total phenolic content (TPC) of aqueous extracts was determined using the Folin–Ciocalteu method, as described by Singleton et al. [30]. Extracts were mixed with diluted Folin–Ciocalteu reagent, diluted 1:10 with distilled water, and incubated in the dark for 5 min. After adding 0.7 M sodium carbonate (Na_2_CO_3_), the mixture was incubated at room temperature for 2 h in the dark. Absorbance was measured at 760 nm, and the results were expressed as milligrams of gallic acid equivalents per gram of product (mg GAE/g), based on a calibration curve of gallic acid standard.

Total flavonoid content (TFC) was determined using the aluminum chloride colorimetric method [31]. Aqueous extracts (100 µL) were first mixed with 60 µL of 5% NaNO_2_ and incubated for 5 min to initiate the reaction. A total of 60 µL of 10% AlCl₃ was then added, and the mixture was incubated for an additional 6 min. The reaction was neutralized with 200 µL of 1 M NaOH, distilled water was added to reach the final volume of 1 mL, and the mixture was incubated for 30 min. Absorbance was measured at 430 nm, and the results were expressed as milligrams of catechin equivalents (CE) per gram of product using a catechin calibration curve.

### 2.5. Short-Chain Fatty Acid Measurement

Short-chain fatty acids (SCFAs), including acetate, propionate, valerate, isovalerate, and butyrate, along with lactate and 3-hydroxybutyrate, were quantified in whole-wheat flour supernatants using hydrazone derivatives. Aqueous supernatants (10 µL) were spiked with 1 µL of a carbon-labeled SCFA internal standard mix containing ^13^C_2_-acetate, ^13^C_3_-propionate, and ^13^C_4_-butyrate (final concentration 0.1 mM). Proteins were precipitated by adding 60 µL of 75% (*v*/*v*) methanol. Derivatization was achieved by mixing the suspension with 60 µL of 200 mM 3-NPH and 10 µL of EDC (120 mM in 6% pyridine). The reaction was incubated for 45 min, at room temperature, under gentle orbital shaking. The derivatization process was stopped by adding 10 µL of 200 mM quinic acid. The mixture was centrifuged at 18,000 rcf for 5 min, at 4 °C, and the resulting supernatants were diluted to 1 mL with 10% (*v*/*v*) methanol. Samples were centrifuged again under the same conditions, and the supernatants were analyzed without further dilution using liquid chromatography–high-resolution mass spectrometry (LC-HRMS). Quantification of SCFA hydrazone derivatives was performed using a U-HPLC system (Ultimate 3000 RS, Thermo Fisher Scientific, Bremen, Germany) coupled to a linear ion trap–Orbitrap mass spectrometer (LTQ Orbitrap XL, Thermo Fisher Scientific). Chromatographic separation was carried out on a core–shell C18 column (Kinetex C18 PS, 100 × 2.1 mm, 2.6 µm; Phenomenex, Torrance, CA, USA), maintained at 40 °C, with mobile phases consisting of water (solvent A) and acetonitrile (solvent B) at a flow rate of 0.2 mL/min. The gradient program for solvent B was as follows: 0 min (5%), 5 min (5%), 12.3 min (35%), 13.3 min (85%), 14 min (99%), and 16 min (99%).

An electrospray ionization (ESI) source operating in negative ion mode scanned the ion range from *m*/*z* 100 to 400 with a resolution of 30,000 (FWHM at *m*/*z* 200). The capillary temperature was set at 300 °C, with sheath and auxiliary gas flow rates at 25 and 15 arbitrary units, respectively. Data acquisition in full MS mode was conducted using Xcalibur 2.1 software (Thermo Fisher Scientific). Quantification was performed using calibration curves prepared with the internal standard technique, covering a linearity range of 0.001–1 mM.

### 2.6. Determination of In Vitro Antioxidant Activities

The antioxidant activity of the extracts was assessed using the DPPH, oxygen radical absorbance capacity (ORAC), and Ferric Reducing Antioxidant Power (FRAP) assays.

In the first assay, a methanolic solution of DPPH (60 µM) was added to various concentrations of the extract, and the mixture was shaken in the dark for 30 min. Absorbance was measured at 517 nm using a spectrophotometer. The antiradical activity (ARA) was calculated using the following formula:

ARA = [1 − (AS/ADPPH)] × 100

where AS is the absorbance of the sample and ADPPH is the absorbance of the DPPH solution. The concentration of the extract required to inhibit 50% of the DPPH radicals (EC50) was determined as described by Colosimo et al. [32].

The ORAC assay was performed as described by Chiellini et al. [33]. Aqueous dilutions of the fermented and non-fermented samples were incubated with fluorescein, used as the fluorescent probe, and AAPH, the radical generator, at 37 °C. Fluorescence decay was measured at an excitation wavelength of 485 nm and an emission wavelength of 514 nm using a Victor™ X3 plate reader (Waltham, MA, USA). Trolox, a vitamin E analog, was used as the reference standard for calibration. ORAC values were expressed as micromoles of Trolox equivalents (µMTE) per 100 g of product using the following formula:

ORAC = (Sample Area − White Area)/(Trolox Area − White Area) × k × a × h

where k is the dilution factor of the sample, a is the concentration of Trolox (µM), and h is the sample concentration (mg/mL).

The FRAP assay was conducted as outlined by Colosimo et al. [32]. Aqueous extracts (33 µL) were mixed with 967 µL of a freshly prepared FRAP reagent containing 10 mM TPTZ dissolved in 40 mM HCl, 20 mM FeCl_3_·6H_2_O, and 300 mM acetate buffer (pH 3.6) in a ratio of 1:1:10. The mixture was gently vortexed to ensure thorough mixing and then incubated at room temperature for 6 min. After 30 min, the absorbance of the samples was measured at 593 nm using a spectrophotometer. The antioxidant activity was quantified and expressed as Fe^2^⁺ equivalents (µM), calculated from a calibration curve prepared with FeSO_4_·7H_2_O ranging from 100 to 2000 µM.

### 2.7. Determination of Ex Vivo Antioxidant Activity

The cellular antioxidant activity in red blood cells (CAA-RBC) assay was used to evaluate the antioxidant potential of the extracts, utilizing human erythrocytes (RBCs) as a biological model according to Blasa et al. [34]. Blood samples from healthy patients (provided by the Monasterio Foundation, San Cataldo-CNR Hospital, Pisa) were collected in EDTA-treated tubes and centrifuged at 2140 rcf for 10 min, at 4 °C, to remove plasma, platelets, and the buffy coat. The isolated RBCs were diluted 1:100 in phosphate-buffered saline (PBS, pH 7.4). A 2 mL aliquot of this suspension was mixed with 250 µL of DCFH-DA, a fluorescent probe (15 µM final concentration), and 250 µL of the extract (1 mg/mL final concentration) or Trolox (500 µM), which was used as the standard compound. The mixture was incubated in the dark for 1 h, allowing the fluorescent probe and test compounds to penetrate the erythrocyte membrane. After incubation, the erythrocytes were washed twice with PBS to remove residual compounds and resuspended in 1 mL of PBS. A radical generator, AAPH (20 µL, final concentration 1.2 mM), was added to 180 µL of the cell suspension to induce oxidative stress. Fluorescence decay was measured using a Victor™ X3 plate reader (Waltham, MA, USA), with readings taken at an excitation wavelength of 485 nm and an emission wavelength of 535 nm.

The antioxidant activity was calculated and expressed as CAA units using the following formula:

CAA unit = 100 − (Area C/Area S × 100)

where Area C is the area under the fluorescence decay curve for the sample, and Area S is the area under the curve for the standard (Trolox).

### 2.8. Statistical Analysis

The results are expressed as the mean ± standard deviation (SD) of three different experiments. Unpaired *t*-test and one-way analysis of variance (ANOVA) with Tukey’s *post hoc* test were used for comparisons; *p*-values < 0.05 were considered statistically significant, with * *p* < 0.05, ** *p* < 0.01, *** *p* < 0.001, and **** *p* < 0.0001. All analyses were carried out using GraphPad Prism version 8.0.

## 3. Results

### 3.1. Selection of Combination of Lactic Acid Bacteria and Yeast for Fermentation of Whole-Wheat Flour

Table 2 shows the outcomes of a screening process designed to identify the optimal combination of yeasts and lactic acid bacteria (Mix) and enhance the nutritional and functional properties of fermented whole-wheat flour. The results indicate that fermentation significantly improved polyphenol and flavonoid content, as well as antioxidant activity (measured by DPPH EC_50_), after 4 days of incubation at 37 °C. Compared to the unfermented flour (1.01 ± 0.05 mg GAE/g dw), all fermented samples exhibited an increase in polyphenols, with Mix 8 showing the highest level (3.40 ± 0.04 mg GAE/g dw), representing a 3.37-fold enhancement. Similarly, flavonoids significantly increased in all Mix fermentations, with Mix 6 showing the greatest improvement (3.25 ± 0.01 mg CE/g dw), a 2.64-fold increase relative to the unfermented control (1.23 ± 0.24 mg CE/g dw), which could be correlated to the proteolytic activity of *E. faecium* and *S. thermophilus*, but without statistically significant differences from that of Mix 7. Antioxidant activity, assessed by the DPPH (EC_50_) assay, demonstrated a notable reduction in EC_50_ values across all fermented samples, indicating enhanced free radical scavenging capacity. Mix 8 exhibited the most significant effect, with an EC_50_ value of 1.01 ± 0.17 mg/mL, representing over a 10-fold improvement compared to the unfermented control (10.87 ± 0.48 mg/mL), and a strong antiradical capacity with respect to the other Mix mixtures (*p* < 0.001). On this basis, Mix 8 was selected for subsequent studies to further explore its effectiveness in enhancing the bioactive compound profile and biological properties of fermented wheat products, as well as its potential applications in food science and nutrition.

### 3.2. Quantification of Short-Chain Fatty Acids

**Figure 1:**
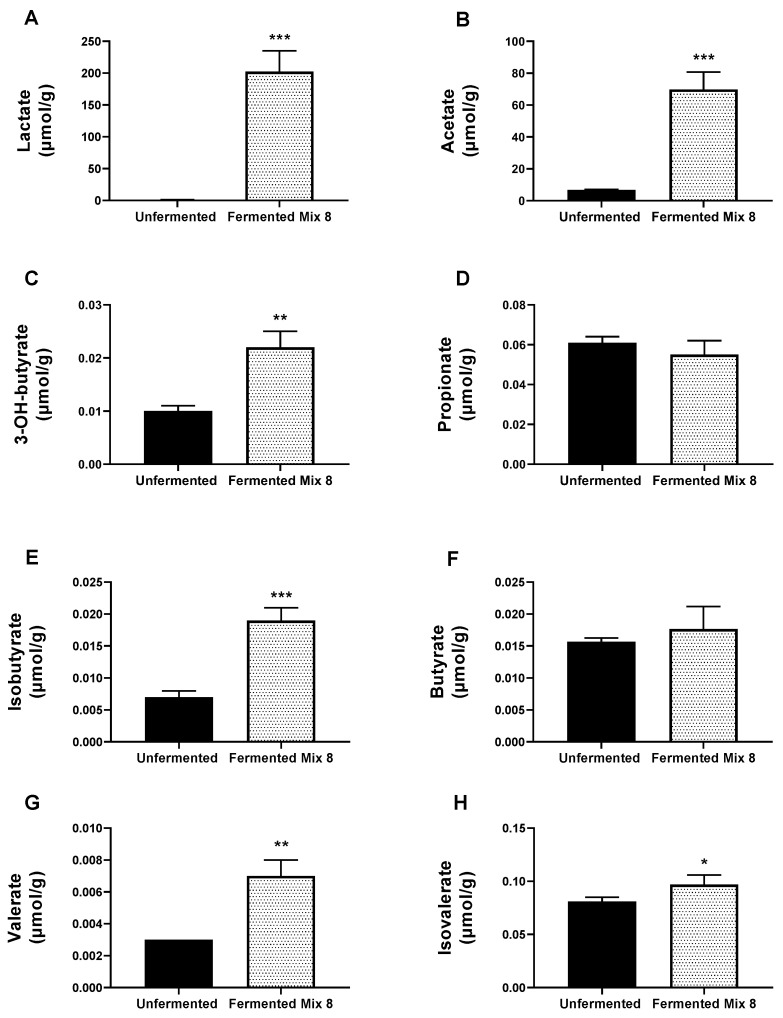
Quantification of short-chain fatty acids in unfermented and fermented whole-wheat flour with Mix 8. Unpaired *t*-test; * Significance versus unfermented whole-wheat flour, with * *p* < 0.05, ** *p* < 0.01, and *** *p* < 0.001. Panels (**A**–**H**) show quantitative results (µmol/g) referring to lactate, acetate, 3-OH-butyrate, propionate, isobutyrate, butyrate, valerate and isovalerate, respectively.

Figure 1 outlines the effects of fermentation with Mix 8 on the short-chain fatty acid (SCFA) and lactate profiles in whole-wheat flour, revealing significant metabolic changes induced by microbial activity. The lactate level in fermented flour strongly increased 217-fold compared to unfermented flour, establishing lactate as the primary fermentation product. Acetate concentration also exhibited a substantial 10.2-fold increase, making it the dominant SCFA produced during fermentation. Among minor SCFAs, 3-hydroxybutyrate increased 2.2-fold, while butyrate levels remained relatively unchanged. Propionate concentration showed no significant differences between fermented and unfermented samples, whereas iso-butyrate levels increased 2.7-fold. Valerate concentration rose 2.3-fold, and isovalerate exhibited a modest but statistically significant 1.2-fold increase.

### 3.3. In Vitro Antioxidant Activities

**Figure 2:**
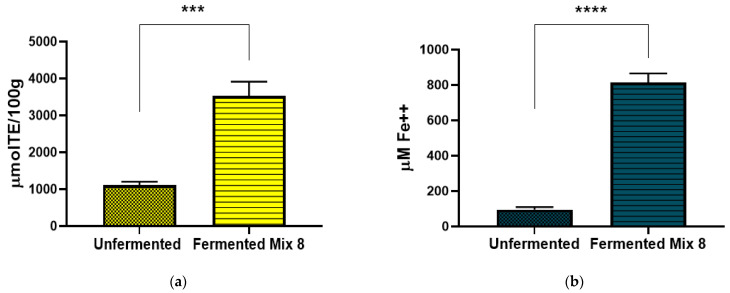
Antioxidant capacity of unfermented and fermented whole-wheat flour with Mix 8. (**a**) ORAC assay expressed as µmol Trolox equivalent (TE)/100 g of dry weight (dw); (**b**) FRAP assay expressed as Fe^2+^ equivalents (µM). Results are expressed as mean ± SD (n = 3). Unpaired *t*-test, with *** *p* < 0.001 and **** *p* < 0.0001.

Figure 2a (ORAC) and Figure 2b (FRAP) present additional evaluations of the antioxidant capacity of unfermented and fermented whole-wheat flour with Mix 8, complementing the findings of the DPPH assay and providing a more comprehensive assessment of the antioxidant potential of this product. Both assays revealed significant enhancements in antioxidant activity following fermentation. The ORAC value of the fermented sample increased 3.2-fold, from 1110.21 ± 90.30 to 3527.30 ± 389.05 µmolTE/100 g, reflecting a marked enhancement in its ability to neutralize peroxyl radicals. Similarly, the FRAP value of the fermented product increased approximately 8.5-fold, from 95.75 ± 14.20 to 813 ± 52.81 µM Fe^2+^, indicating a substantial improvement in its electron-donating and reducing capacity.

### 3.4. Cellular Antioxidant Activity in Red Blood Cells

**Figure 3:**
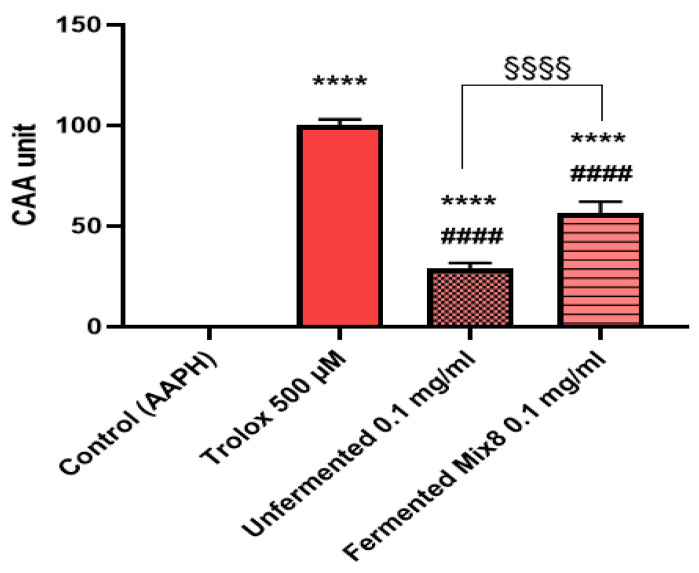
Cellular antioxidant activity (CAA) on red blood cells (RBCs) of unfermented and fermented whole-wheat flour extracts with Mix 8 under oxidative conditions (AAPH). Trolox was used as standard. One-way ANOVA with Tukey’s multiple comparisons test. **** Significantly different from control, AAPH-treated cells (CAA unit = 0); ^####^ significantly different from Trolox; ^§§§§^ significantly different from unfermented flour. Data are expressed as mean ± SD (n = 3) and obtained from blood samples of healthy and distinct volunteers.

Figure 3 presents the results of the CAA-RBC assay, evaluating the cellular antioxidant activity under AAPH-induced oxidative stress in human red blood cells for the unfermented and fermented wheat extracts with Mix 8; Trolox was used as reference standard. The control sample (treated with AAPH alone) showed 0 CAA units, serving as a baseline. The positive control, Trolox (500 µM), achieved 100 ± 3.04 CAA units, validating the assay’s sensitivity. The unfermented whole-wheat extract (0.1 mg/mL) exhibited moderate antioxidant activity with 29.00 ± 2.64 CAA units, significantly lower than both the fermented sample and Trolox. In contrast, the fermented extract (0.1 mg/mL) showed a substantial improvement, achieving 56.43 ± 5.80 CAA units, which is more than double the activity of the unfermented extract (**** *p* < 0.0001).

## 4. Discussion

The present study aimed to select lactic acid bacteria and yeast strains to develop starter cultures able to enhance the functional properties of fermented whole-wheat. To achieve this, targeted microbial combinations, including *P. pentosaceus*, *F. sanfranciscensis*, *L. plantarum*, *L. mesenteroides*, *S. thermophilus*, *E. faecium*, and K. *humilis,* were formulated and applied to whole-wheat fermentation. The resulting fermented samples were comprehensively analyzed for their polyphenol and flavonoid content, as well as their antioxidant activity. All the strains utilized in this study have been extensively documented in the literature for their metabolic performance including the formation of polyphenols and flavonoids as well as the ability to improve antioxidant activity of fermented foods when assessed as single cultures [35,36,37]. Therefore, our results can be due to the antagonistic or synergistic interactions between microbial consortia based on the metabolism of varies molecules including carbohydrates, proteins, and polyphenols during fermentation [17,37]. The improvement in bioactive compounds such as polyphenols and flavonoids after fermentation can be attributed to several factors related to the enzymatic profiles of microbial consortia. *F. sanfranciscensis* is the predominant LAB in traditionally fermented sourdoughs [38,39]. This strictly heterofermentative LAB species establishes a trophic mutualistic relationship with *K. humilis*, a maltose-negative yeast. This nutritional interaction is characterized by the yeast’s ability to hydrolyze sucrose and glucofructans through invertase activity, utilizing glucose as an energy source while releasing fructose and amino acids. Fructose serves as an alternative external electron acceptor for *F. sanfranciscensis*, whereas amino acids complement the LAB species’ metabolic requirements. Both microorganisms adapt to the stresses they impose on each other. *F. sanfranciscensis* generates an acidic environment by producing acetate instead of ethanol, while *K. humilis* induces oxidative stress through thiol oxidation. These stress factors are counterbalanced by redox homeostasis mechanisms and acetic acid tolerance, which occurs without triggering sporulation in the yeast [40,41]. The study of Rogalski and colleagues highlights that *F. sanfranciscensis* has a commensal interaction with *K. humilis* and a competitive interaction with *S. cerevisiae* [42]. Specifically, *K. humilis* produces ethanol, organic acids, and CO_2_, which stimulate LAB growth and enzymatic activity, leading to the release of fiber-bound polyphenols [43,44]. The enzymatic activity of key microbes like *F. sanfranciscensis* and *P. pentosaceus,* present in Mix 3, Mix 5, and Mix 8, facilitates the release of matrix-bound polyphenols through β-glucosidase and esterase activity, breaking down glycosidic and ester bonds in the wheat matrix [45,46,47]. Furthermore, in Mix 6, the addition of *E. faecium* and *S. thermophilus* increases the content of polyphenols, in particular flavonoids, compared to Mix 4. *E. faecium* and *S. thermophilus* are proteolytic bacteria already known for their ability to hydrolyze proteins from different substrates and release bioactive peptides with different activities. *E. faecium* has been isolated from human milk and has been used in the production of various fermented foods, mainly because it plays an important role in the development of organoleptic characteristics [48]. Although there is no evidence for cereals, Cuvas-Limon and colleagues [49] found an increase in total phenolic content in *Aloe vera* fermented by probiotic *E. faecium*.

The specific composition of Mix 8, which includes *K. humilis*, *F. sanfranciscensis, E. faecium*, *P. pentosaceus*, and *L. mesenteroides,* exhibits a better antiradical activity, measured by DPPH compared to other formulations. The production of glucansucrases by *Leuconostoc* strains catalyzes the transfer of glucose to a wide range of non-carbohydrate molecules containing hydroxyl groups, including benzenediols, phenolic acids, and flavonoids, improving their stability and antioxidant activity [50,51].

Our results reveal significant metabolic shifts in short-chain fatty acids (SCFAs) and lactate levels following fermentation with Mix 8. The fermentation of wheat flour induces significant biochemical transformations, particularly a marked increase in the production of lactate and acetate, driven predominantly by the metabolic activity of LAB [37,52]. Through homo- and heterofermentative pathways, LAB convert carbohydrates into lactate and acetate, creating an acidic environment that enhances the solubility and bioavailability of polyphenols while contributing to the sensory and preservative qualities of the product [53]. Lactate acts as a natural preservative by inhibiting spoilage microorganisms and pathogenic bacteria. In the human gut, it also serves as a precursor for butyrate synthesis, promoting the health of the intestinal epithelium and reducing inflammation [54]. Acetate, another major fermentation product, contributes to the product’s flavor profile and exhibits antimicrobial properties, enhancing food safety [55]. It also acts as a systemic energy substrate and regulates lipid metabolism. The increase in this SCFA after fermentation with Mix 8 might offer potential health benefits such as improved metabolic function and reduced cholesterol levels [56,57]. In addition to lactate and acetate, moderate increases in 3-hydroxybutyrate and iso-butyrate were attributed to the microbial metabolism of branched-chain amino acids or intermediates derived from pyruvate. These compounds, along with propionate, butyrate and isovalerate, support gut microbial diversity, with 3-hydroxybutyrate providing neuroprotective benefits and acting as an alternative energy source, especially under conditions of low glucose availability. These SCFAs are well recognized for their health-promoting effects, including glycemic regulation, antioxidant and anti-inflammatory properties, and maintenance of colonic epithelial integrity [58,59].

The increase in antioxidant activity in fermented wheat, as evidenced by DPPH, FRAP, and ORAC assays, could be a consequence of multiple factors related to microbial metabolism and fermentation-driven biochemical transformations [32]. The production of antioxidant metabolites by microorganisms, including *Lactobacillus* spp., *K. humilis*, and *P. pentosaceus*, plays a central role, as these microbes synthesize flavonoids, peptides, and exopolysaccharides with potent free radical scavenging properties [37,60]. Additionally, fermentation-induced chemical modifications, such as decarboxylation, methylation, dihydroxylation, and deglycosylation of phenolic compounds, enhance their redox potential and efficiency in neutralizing free radicals [61]. The acidic environment generated during fermentation by organic acids (e.g., lactic and acetic acids) stabilizes antioxidant compounds and increases their activity by optimizing their structural conformation as electron donors [62].

The enhanced cellular antioxidant activity observed in erythrocytes treated with fermented wheat samples was primarily attributed to the increased bioavailability and efficient cellular uptake of bioactive compounds generated during fermentation. Fermentation processes enzymatically release protein and fiber-bound polyphenols, such as ferulic acid and *p*-coumaric acid, from the wheat matrix, boosting their bioavailability [63]. Both hydroxycinnamic acid derivatives are acid-stable and their chemical reactivity is heightened in the presence of organic acid production typical of the fermentation pathways here described. This environment not only facilitates the structural optimization of these compounds for membrane transport but also enhances their compatibility with the phospholipid bilayers of erythrocytes, allowing for efficient cellular uptake [62]. Once internalized, such bioactive compounds scavenge intracellular reactive oxygen species (ROS) and interact with endogenous antioxidant systems, such as glutathione, catalase, and glyoxalase pathways, to restore cellular redox balance. Focusing on bioactive compounds and cell membrane interaction, fermentation produces other secondary metabolites, including flavonoids and hydroxybenzoic acids, with high membrane permeability and potent antioxidative properties, further amplifying the cellular antioxidant response [60,64]. Structural biochemical modifications of polymerized structures through enzymatic activity increase their ability to neutralize oxidative stress with an overall upregulation of the redox potential [60]. These combined processes could explain the superior antioxidant protection observed in erythrocytes treated with fermented wheat, underscoring the functional benefits of fermentation in enhancing antioxidant activity.

## 5. Conclusions

This study highlights the remarkable efficacy of Mix 8, composed of *K. humilis*, *F. sanfranciscensis*, *E. faecium*, *P. pentosaceus*, and *L. mesenteroides*, in improving the nutritional, functional, and bioactive properties of fermented wheat products. Mix 8 showed superior performance in releasing bioavailable polyphenols and other bioactive compounds, resulting in significantly improved antioxidant activities, as evidenced by DPPH, FRAP, ORAC, and CAA-RBC assays. These findings highlight Mix 8′s capacity to optimize antioxidant defense mechanisms and protect cells, particularly erythrocytes, against oxidative stress. Furthermore, fermentation with Mix 8 substantially increased the production of SCFAs, notably lactate and acetate, which are widely recognized for their gut health-promoting properties, thereby adding functional value to the fermented wheat products.

Our study identified an optimal combination of lactic acid bacteria and yeasts capable of producing a fermented flour with promising health benefits. Based on these findings, future research could focus on the activity and interactions of microorganisms during fermentation. Moreover, detailed characterization of specific bioactive molecules and their biochemical transformations could better explain the mechanisms involved in the fermentative process. In addition, how thermal processing affects bioactive compounds will be investigated.

## Figures and Tables

**Table 1:** The composition of different “Mix” mixtures of yeasts and LAB used in the fermentation process.

Fermentation MIX	Yeasts	Lactic Acid Bacteria
Mix 1	*Saccharomyces cerevisiae* *Kazachstania humilis* ITEM 19255	*Fructilactobacillus sanfranciscensis* ITEM 19254
Mix 2	*Kazachstania humilis* ITEM 19255	*Fructilactobacillus sanfranciscensis* ITEM 19254 *Lactiplantibacillus plantarum* VS513
Mix 3	*Kazachstania humilis* ITEM 19255	*Fructilactobacillus sanfranciscensis* ITEM 19254 *Pediococcus pentosaceus* ITEM 18337
Mix 4	*Kazachstania humilis* ITEM 19255	*Fructilactobacillus sanfranciscensis* ITEM 19254 *Streptococcus thermophilus* E
Mix 5	*Kazachstania humilis* ITEM 19255	*Fructilactobacillus sanfranciscensis* ITEM 19254 *Pediococcus pentosaceus* ITEM 18337 *Lactiplantibacillus plantarum* VS513
Mix 6	*Kazachstania humilis* ITEM 19255	*Fructilactobacillus sanfranciscensis* ITEM 19254 *Enterococcus faecium* ITEM 19253 *Streptococcus thermophilus* E
Mix 7	*Kazachstania humilis* ITEM 19255	*Fructilactobacillus sanfranciscensis* ITEM 19254 *Enterococcus faecium* ITEM 19253 *Lactiplantibacillus plantarum* VS513 *Streptococcus thermophilus* E
Mix 8	*Kazachstania humilis* ITEM 19255	*Fructilactobacillus sanfranciscensis* ITEM 19254 *Enterococcus faecium* ITEM 19253 *Pediococcus pentosaceus* ITEM 18337 *Leuconostoc mesenteroides* GP19

**Table 2:** The effect of fermentation with different “Mix” mixtures on total polyphenol and flavonoid contents and the antiradical activity (DPPH) of whole-wheat flour. Total polyphenol and flavonoid contents are expressed as milligrams of gallic acid equivalent per grams of dry weight (mg GAE/g dw) and milligrams of catechin equivalent per grams of dry weight (mg CE/g dw), respectively. The DPPH values were expressed as EC_50_. One-way ANOVA with the multiple range test was used. The results are expressed as mean ± SD (n = 3).

Fermented Whole-Wheat Flour	Polyphenols mgAGE/g dw	Flavonoids mgCE/g dw	DPPH (EC_50_) mg/mL
Unfermented	1.01 ± 0.05	1.23 ± 0.24	10.87 ± 0.48
Mix 1	2.37 ± 0.13 ***	1.91 ± 0.07 *	3.49 ± 0.24 ****
Mix 2	2.41 ± 0.18 ****	2.31 ± 0.26 ***	2.87 ± 0.18 ****
Mix 3	2.54 ± 0.24 ****	2.32 ± 0.0.26 ***	2.31 ± 0.02 ****
Mix 4	2.26 ± 0.08 ***	1.77 ± 0.04 *	4.21 ± 0.30 ****
Mix 5	2.44 ± 0.18 ****	2.04 ± 0.04 **	2.54 ± 0.04 ****
Mix 6	2.92 ± 0.12 ****	3.25 ± 0.01 ****	3.15 ± 0.05 ****
Mix 7	3.13 ± 0.18 ****	2.68 ± 0.08 ****	2.48 ± 0.45 ****
Mix 8	3.40 ± 0.04 ****	2.55 ± 0.17 ***	1.01 ± 0.17 ****

* Significance versus unfermented whole-wheat flour; * *p* < 0.05, ** *p* < 0.01, *** *p* < 0.001, and **** *p* < 0.0001.

## Data Availability

The original contributions presented in the study are included in the article, further inquiries can be directed to the corresponding author.

## Supplementary Materials

The following supporting information can be downloaded at: https://www.mdpi.com/article/10.3390/foods14030421/s1, Table S1: List of primer used in the identification of bacterial and yeast strains and their PCR conditions.
