# Supplementary material for: Fermentation of Whole-Wheat Using Different Combinations of Lactic Acid Bacteria and Yeast: Impact on In Vitro and Ex Vivo Antioxidant Activity
Source: Foods. 2025 Jan 28;14(3):421. doi: 10.3390/foods14030421 (PMC11816817; doi:10.3390/foods14030421)
Supplement: Supplementary file 1 [file foods-14-00421-s001.zip › foods-3383940-supplementary.pdf]

**Table S1.** List of primer used in the identification of bacterial and yeast strains and their PCR conditions.

| Target genes | Primer | Primer sequence (5′-3′) | PCR conditions | References |
|---|---|---|---|---|
| 16S rRNA sequencing | p8FPL | AGTTTGATCCTGGCTCAG | a | [26] |
| | p806R | GGACTACCAGGGTATCTAAT | | |
| 26S rDNA sequencing | NL1 | GCATATCAATAAGCGGAGGAAAAG | b | [27] |
| | NL4 | GGTCCGTGTTTCAAGACGG | | |
| ITS region sequencing | ITS5 | TCCTCCGCTTATTGATATGC | c | [28] |
| | ITS4 | GGAAGTAAAAGTCGTAACAAGG | | |

PCR conditions

| | |
|---|---|
| a | Initial denaturation at 95 °C for 5 min; 30 cycles of 95 °C for 1 min, 55 °C for 1 min, and 72 °C for 2 min; final extension at 72 °C for 5 min |
| b | Initial denaturation at 94 °C for 5 min; 36 cycles of 94 °C for 1 min, 52 °C for 20 s, and 72 °C for 2 min; final extension at 72 °C for 7 min |
| c | Initial denaturation at 94 °C for 2 min; 27 cycles of 96 °C for 10 s, 50 °C for 5 s, and 60 °C for 4 min; final extension at 72 °C for 7 min |

**References**


26. McCabe, K.M.; Khan, G.; Zhang, Y.-H.; Mason, E.O.; McCabe, E.R.B. Amplification of Bacterial DNA Using Highly Conserved Sequences: Automated Analysis and Potential for Molecular Triage of Sepsis. Pediatrics 1995, 95, 165–169, doi:10.1542/peds.95.2.165
27. Kurtzman, C.P.; Robnett, C.J. Identification and Phylogeny of Ascomycetous Yeasts from Analysis of Nuclear Large Subunit (26S) Ribosomal DNA Partial Sequences. Antonie Van Leeuwenhoek 1998, 73, 331–371, doi:10.1023/A:1001761008817
28. Schoch, C.L.; Seifert, K.A.; Huhndorf, S.; Robert, V.; Spouge, J.L.; Levesque, C.A.; Chen, W.; Fungal Barcoding Consortium; Fungal Barcoding Consortium Author List; Bolchacova, E.; et al. Nuclear Ribosomal Internal Transcribed Spacer (ITS) Region as a Universal DNA Barcode Marker for Fungi. Proceedings of the National Academy of Sciences 2012, 109, 6241–6246, doi:10.1073/pnas.1117018109
